# Unraveling the Action Mechanism of Tubeimoside-1 against Tumor Microvessels via Network Pharmacology and Experimental Validation

**DOI:** 10.7150/jca.90391

**Published:** 2024-01-01

**Authors:** YinRong Yang, Qian Wang, FengXia Zhan

**Affiliations:** 1 Department of Clinical Laboratory, Qilu Hospital, Shandong University (Qingdao), Qingdao, Shandong 266035, China.; 2 Department of Clinical Laboratory, Qilu Hospital, Shandong University, Jinan, Shandong 250012, China.; 3 Department of Clinical Laboratory, Shandong University School Hospital, Jinan, Shandong, 250012, China.

**Keywords:** TBMS1, trans-endothelial migration, adhesion protein, tight junction, network pharmacology.

## Abstract

**Objective:** Tubeimoside-1 (TBMS1) is a plant-derived triterpenoid saponin that exhibits pharmacological properties and anti-tumor effects, but the anti-tumor microvessels of action of TBMS1 remains to be completely elucidated. This study aims to verify the effect of TBMS1 on tumor microvessels and its underlying mechanism.

**Methods:** A SKOV3 xenografted mouse model were constructed to evaluate the anti-tumor microvessels of TBMS1 in *vivo*, followed by function assays to verify the effects of TBMS1 on the proliferation, cell cycle, migration, and tubule formation of vascular endothelial cells in *vitro*. Next, based on network pharmacology, the drug/disease-target protein-protein interaction (PPI) networks, biological functions and gene enrichment analyses were performed to predict the underlying mechanism. Finally, molecules and pathways associated with tumor trans-endothelial migration were identified.

**Results:** TBMS1 treatment effectively reduced tumor microvessel density in ovarian cancer model and inhibited the proliferation, cell cycle, migration, and induced apoptosis of vascular endothelial cells *in vitro*. Network pharmacological data suggested that tumor cell adhesion and trans-endothelial migration may participate in antiangiogenic effects of TBMS1. By endothelial adhesion and permeability assay, we identified that tumor adhesion and the permeability of endothelial monolayers were reduced by TBMS1. Furthermore, adhesion protein (VCAM-1and ICAM-1) and tight junction (TJ) proteins (VE-cadhsion, ZO-1 and claudin-5) were found to be regulated. Finally, Akt, Erk1/2, Stat3 and NF-κB signaling were decreased by TBMS1 treatment.

**Conclusion:** To sum up, our findings strongly suggest that clinical application of TBSM1 may serve as a vasoactive drug treatment to suppress tumor progression.

## Introduction

The tumor vasculature is an important component of the tumor microenvironment providing nutrients essential for tumor genesis and development. The hyper-proliferation and migration of vascular endothelial cells (VECs) in tumors results in the formation of chaotic and destabilized vasculature, which leads to limited nutrient delivery, hypoxia, and acidosis, and promotes tumor growth, metastasis, and recurrence 1, 2. Therefore, inhibiting abnormal activity of VECs is a promising anticancer strategy. Otherwise, tumor vessels are abnormal, both structurally and functionally. The inner wall of blood vessels is composed of endothelial cells (ECs) interconnected by junctional molecules. Of which, adhesive molecules expressed at the basolateral surface of activated VECs regulate intravasation of cancer cells 3. Meanwhile, tight junction proteins (TJs) expressed at the tight junction between VECs alter cancer cell migration by controlling the permeability of endothelial monolayers 4. Thus, adhesive molecules and TJs expression of VECs play important roles in tumor trans-endothelial migration.

Tubeimoside-1 (TBMS1) is a major active ingredient of the Chinese medicinal herb *Bolbostemma paniculatum (Maxim) Franquet (Cucurbitaceae)*. TBMS1 exhibits anti-tumor activity in a variety of tumor contexts, including lung cancer, cervical cancer, and ovarian cancer with low toxicity, and usually exerts its anticancer action through the toxicity on cancer cells, including inhibiting proliferation, inducing apoptosis, autophagy, and cycle arrest 5, 6. Although TBMS1 has been reported to inhibit tumor angiogenesis by regulating of angiogenesis-related growth factors and their receptors 7, the anti-tumor microvessels of action of TBMS1 remains to be completely elucidated.

The main purpose of this study was to investigate the mechanism of action and impact of TBMS1 on tumor microvessels, we performed *in vivo* mouse tumor models and *in vitro* cell activity assay and then analyzed and predicted that potential targets and pathways of TBMS1 using network pharmacology technology. Here, we found that TBMS1 suppressed tumor microvessel density in tumor models, and acted as a potent regulator of vascular activity that targets VECs to counteract abnormal tumor adhesion and vascular permeability. In conclusion, TBMS-1 acts as a vasoactive drug to preserve vascular integrity to inhibit tumor cells trans-endothelial migration.

## Materials and methods

### Cell lines and reagents

Human ovarian cancer cell SKOV3, and human umbilical vein endothelial cell HUVEC were maintained in RPMI 1640 medium (Gibco, Waltham, MA, USA), supplemented with 10% fetal bovine serum, 100 U/mL penicillin and 100 mg/mL streptomycin, and kept at 37ºC in a humidified atmosphere containing 5% CO_2_. All cell lines purchased from Shanghai Cell Biology Institute (Shanghai, China). Tubeimoside-1 (TBMS1) was purchased from Yuanye Bio-technology Co. Ltd. (Shanghai, China).

### Xenograft tumor models

Five-week-old female nude BALB/c mice, were purchased from Beijing HFK Bioscience Co., Ltd. (Beijing, PR China). SKOV3 cells (5×10^6^ per mouse) were subcutaneously (*s.c.*) injected into the right flank of nude mice. When the tumor volumes reached about 100 mm^3^, mice were randomized to two groups (n=6) and treated with TBSM-1 (10 mg/kg orally daily for consecutive 14 days) or normal saline (NS). After 14 days, the mice were euthanized. Animal experimental procedures were conducted in accordance with guidelines for experimental animals and approved by the Animal Ethics Committee of Shandong University.

### Immunohistochemistry (IHC)

IHC staining with hematoxylin and eosin (H&E), Ki67, and CD31 (Abcam, Cambridge, UK) of xenograft tumor tissue sections was performed using a DAB substrate kit (Maxin, Fuzhou, China), according to the manufacturer's instructions.

### MTT assay

Cells were seeded in a 96-well plate at a concentration of 5×10^3^ per well and cultured with different concentrations of TBMS1 for 1-3 days. The cell number was evaluated using an MTT assay (Beyotime, Beijing, China). The absorbance value (OD_490_) was obtained using a microplate reader (Synergy 2; BioTek, Winooski, Vermont, USA) at a wavelength of 490 nm.

### Flow cytometry assay

Cells (2×10^5^ per well) were plated into 6-well plate and cultured with different concentrations of TBMS1 for 24 h. Cells were harvested and washed in PBS (phosphate buffered solution). For apoptosis analysis, cells were tested using an apoptosis kit (BestBio, Shanghai, China) and analyzed by flow cytometry (BD Biosciences, CA, USA). For cell cycle analysis, cells were fixed in 70% ethanol at 4ºC overnight. After washing with phosphate-buffered saline (PBS), the fixed cells were incubated in PBS containing 20 μg/mL of propidium iodide (PI), 200 μg/mL of RNase A, and 0.1% Triton X-100 (BD Biosciences, CA, USA) at 37ºC for 30 minutes. The stained cells were then analyzed for cell cycle distribution using a FACSCalibur flow cytometer (BD Biosciences, CA, USA).

### Quantitative real-time (qRT) PCR

Total RNA was extracted using TRIzol reagent (Invitrogen, Carlsbad, California, USA), according to the manufacturer's instructions, and cDNA was synthesized by PrimeScript™ RT Reagent Kit (TaKaRa Biotechnology, Co., Ltd., Dalian, China). Next, SYBR Green PCR Master Mix (TaKaRa Biotechnology) was used to perform real-time-PCR (qPCR). All reactions were carried out on an Applied Biosystems 7500 Real-Time PCR System (Thermo Fisher Scientific, Inc.). Relative gene expression was analyzed using the 2^-ΔΔCt^ method; PCR primer sequences are provided in Table S1.

### Tube formation assay

The tube formation assay was performed using HUVEC cells, as previously described 8. Briefly, 96-well plates pre-coated with 50 μL growth factor reduced (GFR) Matrigel basement membrane matrix (BD Biosciences, CA, USA) were incubated at 37°C for 1 h to allow gel formation. HUVEC cells (3×10^5^ per well) were plated into the plate. Tube formation was assessed after 6 h and photographs were taken using an inverted fluorescence microscope (Olympus, Japan).

### Wound-healing assays

The wound healing assay was performed according to a previously described methodology 9 to test cell migration. Cells (1×10^6^) were spread onto 6-well plates marked at the bottom to the cells reached more than 95% confluency. A 100 μL pipette tip was used to scratch the cells along the marks. The cell debris were washed off with PBS buffer and fresh serum-free medium was added into the plate. Then, cell migration was observed and imaged at 0 and 24 h by using an inverted microscope (Zeiss Axioskop 2, German).

### Transwell assay

The invasion assay was performed using transwell chambers coated with fibronectin and matrigel (8.0 μm pore size; Millipore, MA). Briefly, HUVEC cells (1×10^5^ per well) were added to the upper chamber of transwell filters in a 24-well plate. RPMI 1640 with 10% FBS was added to the lower chamber. Cells were treated with PBS or TBMS1 and incubated for 24 h. Cells that migrated to the bottom of the filter were stained with crystal violet (Solarbio, Beijing, China).

### Endothelial adhesion assay

HUVEC cells (2×10^5^ per well) were seeded in a 12-well plate and pretreated with TBMS1 (10 μg/mL) for 24 h prior to treatment with TNF-α (10 ng/mL) for an additional 6 h. Next, CFSE-labeled SKOV3 or B16 cells (1×10^5^ per well) were added on the top of the VEC monolayers for 2 h. After 2 h, wells were washed gently 3 times with PBS to remove non-adherent cells and adherent cells were photographed with a fluorescent microscope; a minimum of 5 fields/well were quantified.

### Endothelial permeability assay

HUVEC cells (2×10^4^ per well) were added to the upper chamber of a transwell insert (0.4 μm pore size; Millipore) in a 24-well plate. Cells were allowed to reach confluence, and were then treated with PBS or TBMS1 for 24 h. Rhodamine-dextran (10 mg/mL, average mw~70,000; Sigma, USA) was then added to the top well. The appearance of rhodamine-dextran in the bottom well was monitored during a 1 h time course. The absorbance at 590 nm at each time point was recorded.

### Western blot analysis

Proteins from the HUVEC cell lines were extracted using RIPA buffer (BestBio, Shanghai, China) containing protease inhibitor cocktail (Roche Diagnostics). The proteins were then separated by 10% SDS-PAGE and transferred onto PVDF membranes, which were blotted with primary antibodies. Rabbit polyclonal antibodies against p-Akt (Ser473), Akt, p-Erk1/2 (Thr202/Tyr204), Erk1/2, p-Stat3 (Tyr705), p-Stat3 (Ser727), Stat3, p-NFκB (Ser536), NF-κB, and GAPDH were purchased from Cell Signaling technology (Beverly, MA, USA). Membranes were then stained with the appropriate secondary antibody conjugated with HRP, then visualized using enhanced chemiluminescence (Millipore, Billerica, MA, USA), and finally analyzed by ImageLab software (Version 3.0, Bio-Rad).

### Target prediction

SMILES of Tubeimoside-1 were obtained from Pubchem chemical information database (https://pubchem.ncbi.nlm.nih.gov/) and imported into Swiss Target Prediction database (http://www.swisstargetprediction.ch/), limited species for "Homo sapiens" to predict its related targets. Tubeimoside-1-related targets were also predicted using ChEMB (https://www.ebi.ac.uk/chembl/) and Genecards database (https://www.genecards.org/). The intersection of the above databases and the deletion of repeated targets were considered as drug targets. Moreover, the GeneCards (https://www.genecards.org/) were searched with “tumor microvessels” as the keyword to obtained the candidate targets of disease.

### Network construction

All targets were imported into UniProtKB (http://www.uniprot.org/) to unify the target names. VENNY2.1 (https://bioinfogp.cnb.csic.es/tools/venny/) was used to obtain the intersection targets of the compound and disease. The intersecting targets were imported into STRING (https://cn.string-db.org/cgi/input.pl), “Homo sapiens” was selected as the species, and medium confidence >0.4 was selected as the minimum interaction threshold, unlinked targets were hidden, and other parameters were kept at default settings. The tsv file was saved after updating and imported into Cytoscape 3.9.1. Then, network analysis was performed, and the clusters with high correlation were calculated using the CytoNCA plugin.

### GO and KEGG pathway enrichment analysis

The above intersection targets were imported into the Metascape database (https://metascape.org/) and the DAVID v6.8 database (https://david.ncifdrf.gov/), and the species was set as “Homo Sapiens” to conduct the enrichment analysis of biological functions and signaling pathways. The KEGG pathways of p < 0.01 were considered significant, and the results of enrichment analysis were visualized using the microbiology online mapping platform (http://www.bioinformatics.com.cn/).

### Statistical analysis

Each experiment was performed in triplicate, and data are expressed as mean ± SEM, unless otherwise stated. Student's t-test was used to compare mean values. If data were not normally distributed or if they had unequal variances, the Mann-Whitney U test was used for comparison of two groups. All analyses were conducted using GraphPad Prism software 6 (GraphPad Software Inc., La Jolla, CA). A p-value of *p* < 0.05 was considered to indicate a statistically significant difference. ^*^p <0.05, ^**^p <0.01, and ^***^p <0.001.

## Results

### TBSM1 inhibits tumor microvessel density

**Figure 1:**
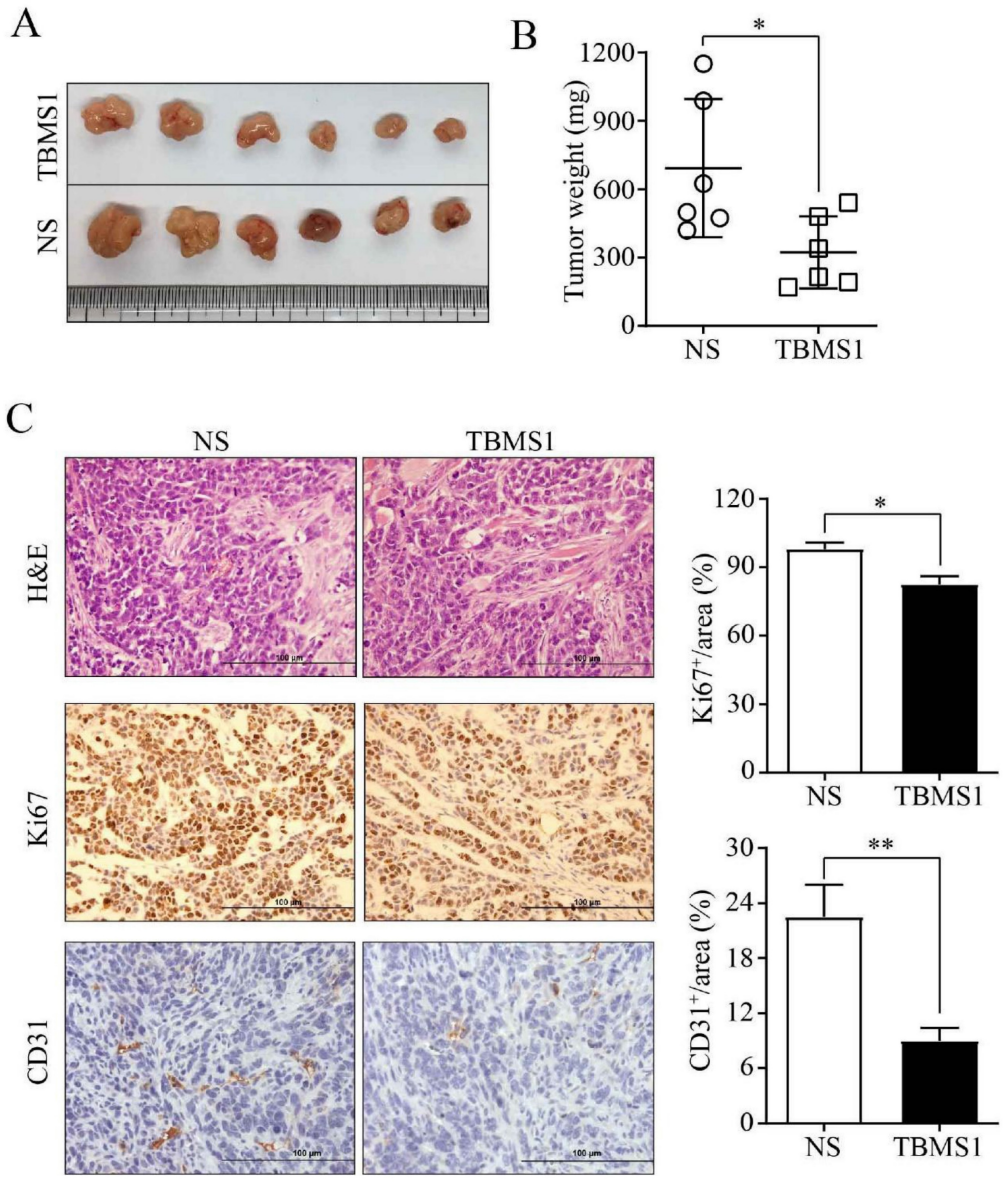
** TBMS1 inhibits tumor growth and angiogenesis in an ovarian cancer xenograft model.** SKOV3 cells were subcutaneously (s.c.) injected of into nude mice. Mice bearing palpable tumors were treated with TBMS1 or normal saline (NS). Tumor tissue (A), statistical analysis of tumor weight (B), representative images of H&E, Ki67, and CD31 staining, and quantification of Ki67 and CD31 (C) in xenograft tumors are shown. The scale bar represents 100 μm. Results are shown as mean ± SEM, n = 6. *p < 0.05, **p < 0.01.

In order to study the anti- tumor microvessels of TBSM1 *in vivo*, the human ovarian cancer cell SKOV3 was subcutaneously injected into the flanks of female nude mice. As the tumors were established, the mice were randomized to receive TBSM1 treatment for 2 weeks. Daily administration of 10 mg/kg/day TBSM1 significantly reduced the volume of the developing tumors (Figure 1A). After therapy, the tumor weight in the TBMS1 and NS control groups was 323 ± 157 mg and 694 ± 304 mg, respectively (Figure 1B). Immunohistochemical analyses of the excised tumors revealed a lower density of Ki-67-stained proliferating cells in the tumors from the TBMS1 group compared to those from the vehicle-treated animals (*P* <0.05) (Figure 1C), indicating that TBSM1 efficiently suppressed tumor growth. Furthermore, the CD31-positive microvessels in the tumors from the TBMS1 group were significantly reduced comparing with vehicle-treated group (*P* <0.01) (Figure 1C), indicating that TBSM1 efficiently suppressed tumor microvessel density (MVD).

### TBSM1 inhibits proangiogenic properties of vascular endothelial cells

**Figure 2:**
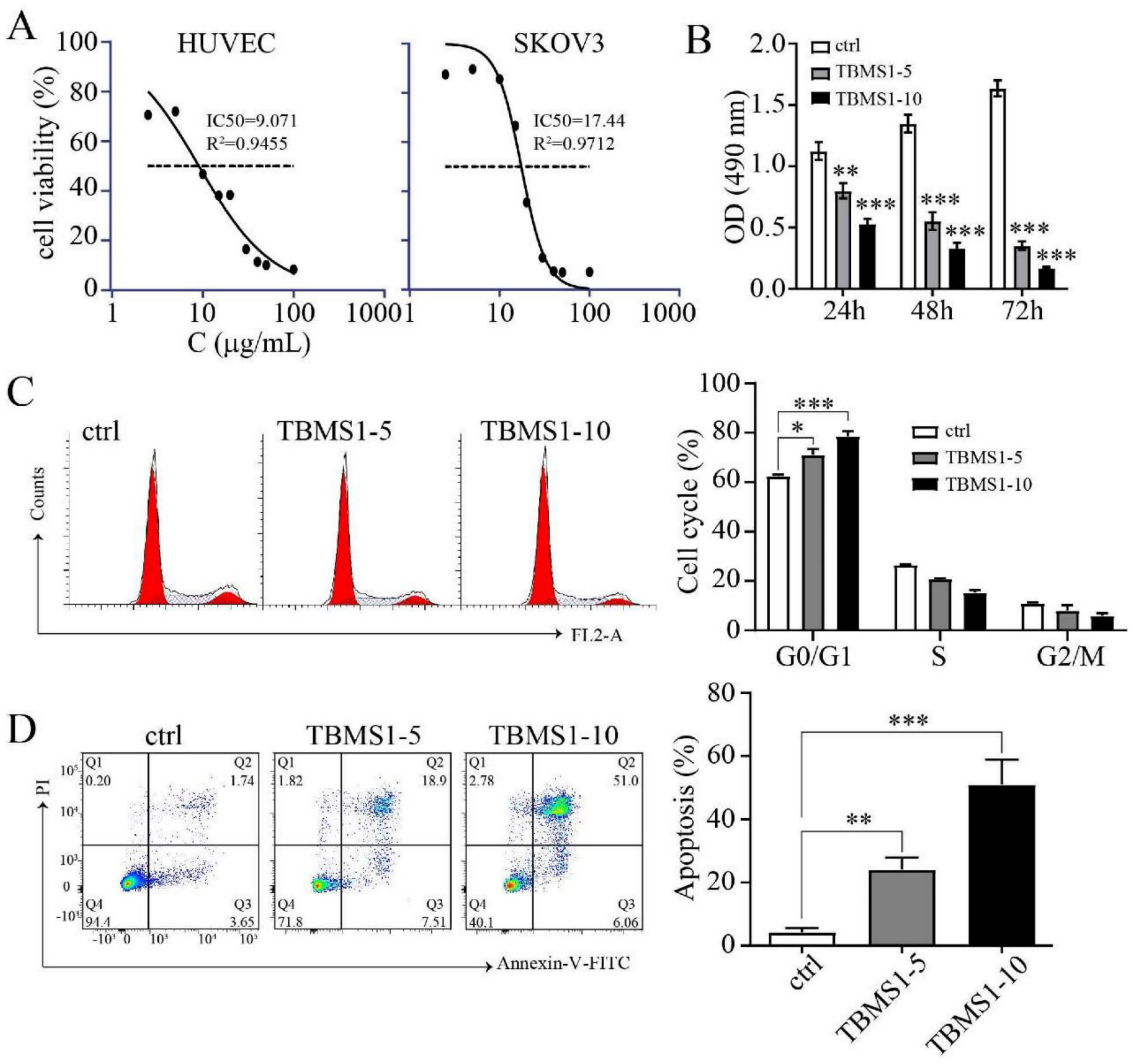
** TBMS1 inhibits the proliferation, cell cycle progression, and promotes apoptosis of HUVEC cells.** (A) HUVEC and SKOV3 cells were treated with TBMS1 (0, 2.5, 5, 10, 15, 20, 30, 40, 50, 100 μg/mL) for 24 h. (B) MTT assay was performed to test cell viability after HUVEC cells were incubated with TBSM-I (5, 10 μg/mL) for 24 h, 48 h, and 72 h. (C-D) Cell cycle and apoptosis rates were measured by flow cytometry after HUVEC cells were treated with TBSM-I (5, 10 μg/mL) for 24 h. The summary data are shown on the right. Data are presented as mean ± SEM of at least three independent experiments. *p < 0.05, **p < 0.01, ***p < 0.001.

In solid tumors, resident vascular endothelial cells (VECs) possess high proangiogenic properties, including increased proliferation and migration 10. To demonstrate the anti-angiogenic effects of TBMS1 on vascular endothelial cell growth, we analyzed the proliferation of HUVEC cells treated with TBMS1. The half-maximal inhibitory concentration (IC_50_) of TBMS1 was higher (17.44 ± 0.75 μg/mL) for ovarian cancer cells than HUVEC cells (9.07 ± 0.95 μg/mL) (Figure 2A), and the anti-proliferative activity of TBMS1 at 10 μg/mL against SKOV3 was less than 15% (data not shown). These results suggested that endothelial cells are more sensitive to TBSM1 treatment than cancer cells. Correspondingly, the proliferation of HUVEC cells treated with TBMS1 was suppressed in a time- and dose-dependent manner (Figure 2B). Inhibition of cell growth is usually associated with cell cycle and apoptosis, and flow cytometric analyses revealed that HUVECs treated with TBMS1 showed a dramatic increase in cell cycle arrest and apoptosis (Figure 2C, D). These results indicate that TBMS1 influences vascular endothelial cell proliferation by causing cell cycle arrest and inducing apoptosis.

**Figure 3:**
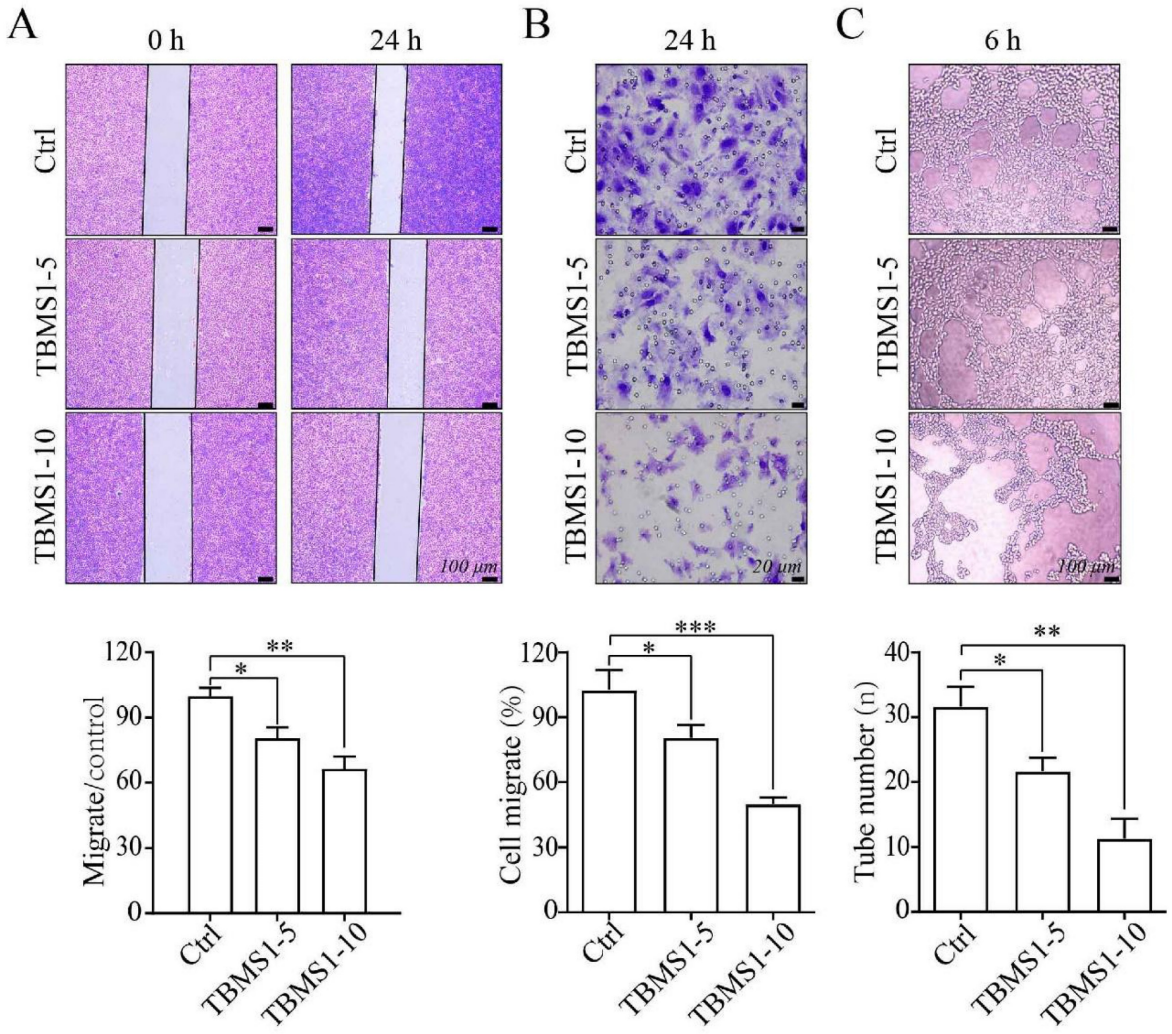
** TBMS1inhibits the cell migration and tube formation of HUVEC cells.** (A-B) HUVEC cells were exposed to TBSM1 (5, 10 μg/mL) for 24 h. HUVEC cell migration was analyzed by wound healing assay (A) and transwell assay (B). (C) Tube formation of HUVEC cells was measured after treatment with TBSM1 (5, 10 μg/mL) for 6 h. Representative images are shown at the top, and summary data are shown at the bottom. Data are presented as mean ± SEM of at least three independent experiments. *p < 0.05, **p < 0.01.

Next, we investigated the effects of TBMS1 on the HUVEC cell migration, using wound healing assays and transwell invasion assays. Treatment of HUVEC cells with TBMS1 for 24 h caused an inhibition of cell migration and invasion compared with control treated cells (Figure 3A and B). Furthermore, an *in vitro* Matrigel model was employed to study tube formation, and TBMS1 treatment resulted in a significant decrease in the numbers of capillary networks (Figure 3C). These data demonstrate that TBMS1 directly inhibits vascular endothelial cell proliferation, migration, and tube formation potential *in vitro*.

### TBSM1 suppresses vascular permeability to inhibit trans-endothelial metastasis

**Figure 4:**
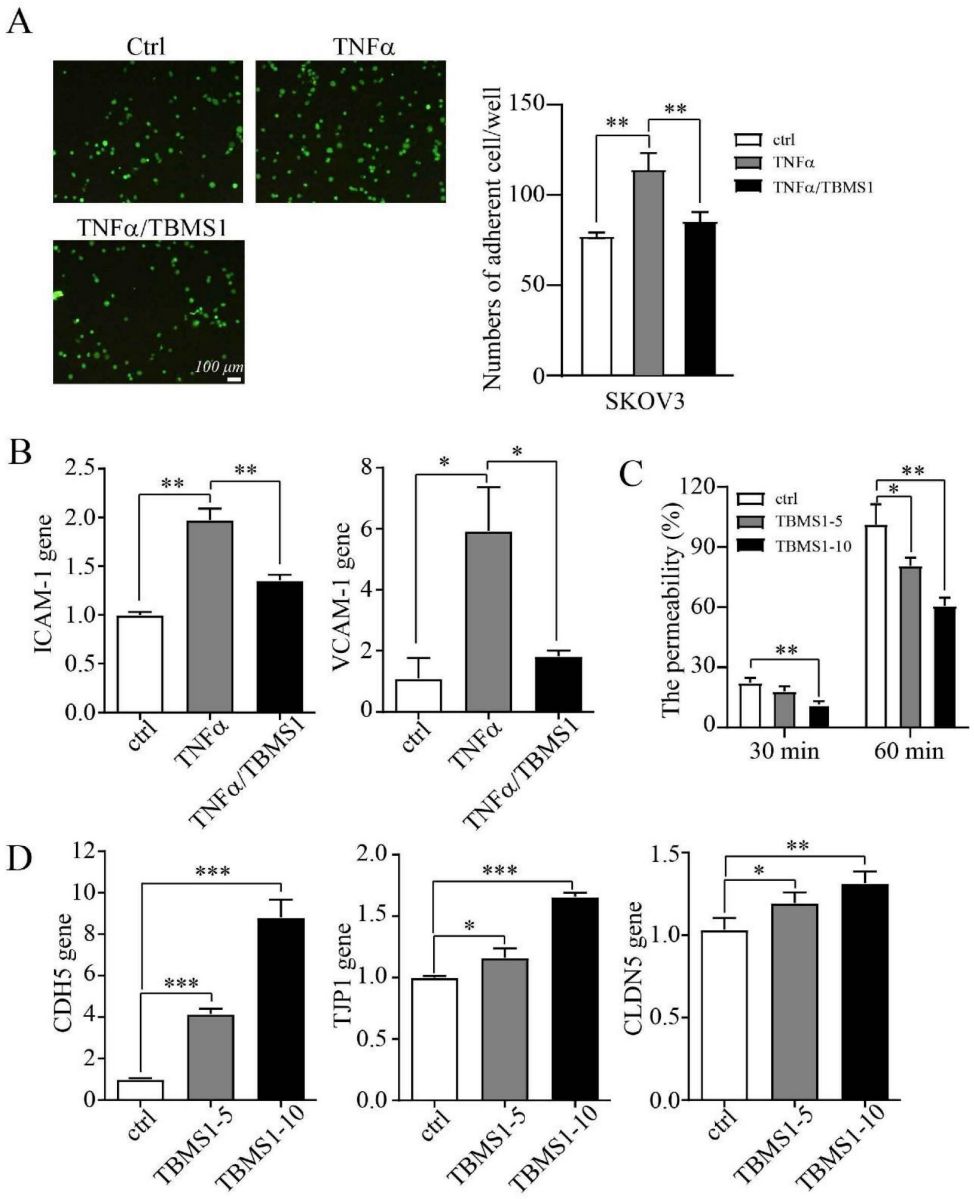
** TBMS1 inhibits tumor cell adhesion and vascular permeability.** (A) Representative micrographs of SKOV3 cells (green) adhering to a VEC monolayer upon treatment with TNF-α (10 ng/mL) or TNF-α plus TBSM-I (10 μg/mL) are shown on the left. The quantification of the number of adherent cells is shown on the right. (B) mRNA expression levels of ICAM-1 and VCAM-1 in VEC monolayer upon treatment with TNF-α (10 ng/mL) or TNF-α plus TBSM1 (10 μg/mL) were analyzed by RT-PCR. (C) The permeability of the VEC monolayer upon treatment with TBSM1 (5, 10 μg/mL) grown on 0.4 μm filters was measured by the appearance of rhodamine-dextran in the bottom well. The absorbance at 590 nm at each time point was indicated. (D) mRNA expression levels of VE-cadherin (CDH5), TJP1 (ZO-1), and claudin-5 (CLDN5) in HUVEC cells after treatment with TBSM1 (5, 10 μg/mL) were analyzed by RT-PCR. Data are presented as mean ± SEM of at least three independent experiments. *p < 0.05, **p < 0.01.

Cancer cells adhering to VECs and subsequent migrating trans- endothelial are considered to be a key step of tumor metastasis, while endothelial barrier posed of many adhesion molecules and tight junction (TJs) proteins plays important roles in this process 11, 12. We queried whether TBMS1 treatment rendered VECs to be less adhesive to cancer cells. Indeed, TBMS1 reduced the number of cancer cells adhering to VECs that were pre-activated with tumor necrosis factor alpha (TNF-α) (Figure 4A), which regulated the expression of adhesive molecules through NF-κB signaling 13. Notably, TBMS1-treated VECs expressed lower levels of the adhesion molecules VCAM-1 and ICAM-1 (Figure 4B), which were involved in cancer cell intra/extravasation 14. Additionally, disorganized tumor vessels express lower levels of junction proteins, which disrupts vascular integrity and facilitates tumor metastasis 15. Therefore, we performed an *in vitro* vascular permeability assay using rhodamine-labeled dextran, as described previously 16. Treatment of the VEC monolayer with TBMS1 reduced the passage of dextran from the top to the bottom wells (Figure 4C). Simultaneously, there was a marked increase of VE-cadherin and a moderate increase of the TJ proteins zonula occludens-1 (ZO-1) and claudin-5 induced by TBMS1 (Figure 4D). These results suggest that TBMS1 suppresses trans-endothelial metastasis by reducing tumor adhesion and restoring the integrity of the endothelial cell barrier.

### The pharmacological mechanisms of TBSM1 against tumor angiogenesis via network pharmacology

**Figure 5:**
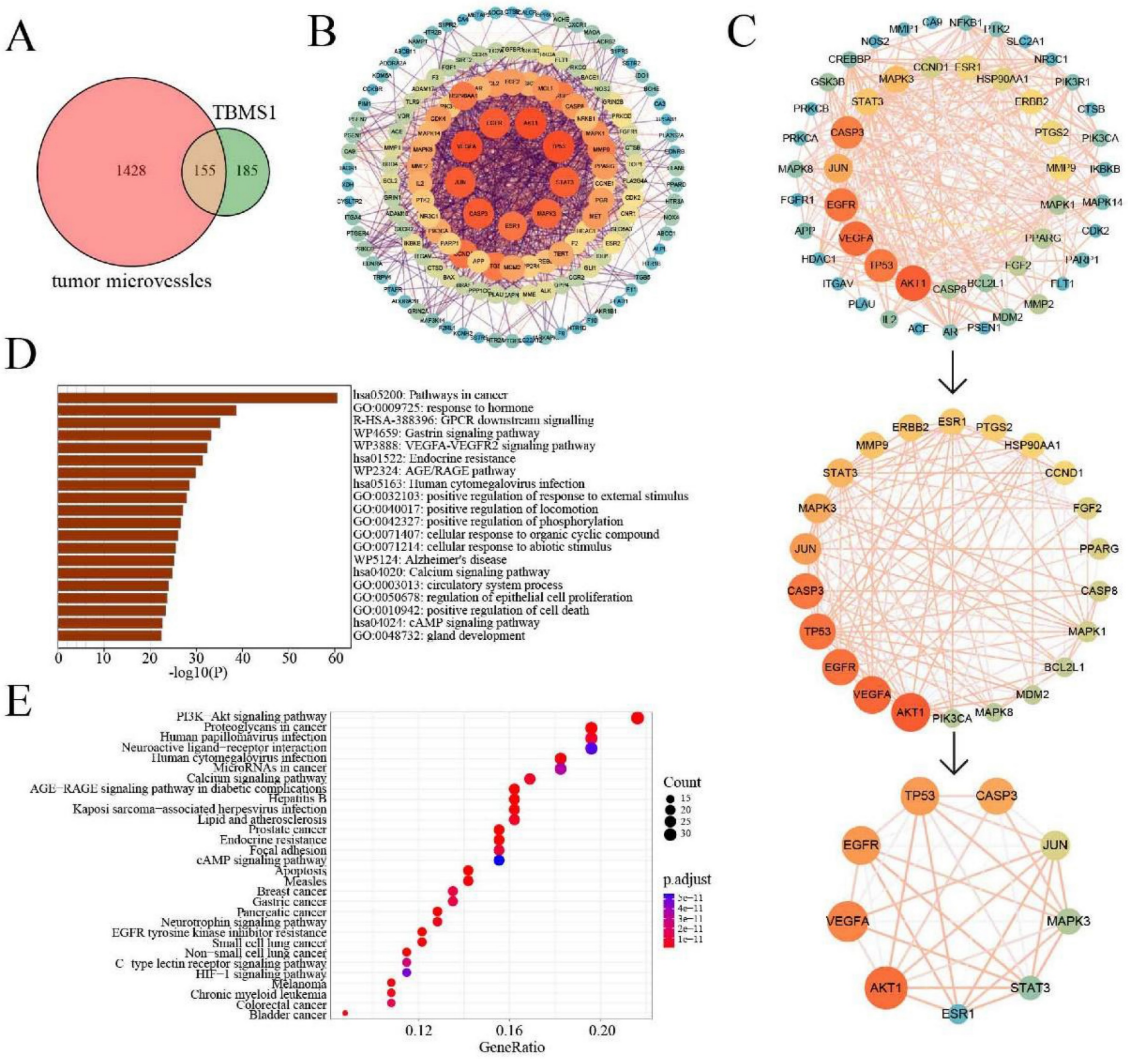
** Network pharmacology analysis of TBMS1 against tumor microvessels.** (A) The Venny results of potential target genes of TBMS1- tumor microvessels. (B) The PPI network map of 155 target genes analyzed by STRING. (C) Key targets in PPI network are screened using the CtyoNCA plugin of Cytoscape. (D-E) The core targets that were enriched different biological processes and signaling pathways as assessed by Metascape (D) and DAVID v6.8 (E) (p-value <0.05).

Through target prediction and database search, a total of 340 potential action targets of TBMS1 and 1,583 tumor microvessels-related targets were obtained. Moreover, 155 intersection targets were screened using online drawing to make a venn diagram (Figure 5A; Table S2). Sequentially, we used STRING to construct the intersection targets of the PPI network, and the results were analyzed using Cytoscape software (version 3.8.0) (Figure 5B). The results showed that 151 nodes and 1819 edges were obtained, and average node degree was 23.5. Furthermore, the top 9 interacting hub genes including AKT1, VEGFA, EGFR, TP53, CASP3, JUN, MAPK3, STAT3 and ESR1, were obtained based on three core networks using the CtyoNCA plugin of Cytoscape (Figure 5C), which was identified as key hub proteins and may play an important role in the efficacy of TBMS1 in the treatment of tumor microvessels. The biological functions (GO-Biological Process, GO-BP) analysis using Metascape indicated that the above targets were mainly related to pathways in cancer, VEGFA-VEGFR2 signaling pathways, and positive regulation of locomotion, phosphorylation and cell death (Figure 5D), and the results of the KEGG analysis using DAVID indicated that the above targets were closely associated with mechanisms such as the PI3K-Akt signaling pathway, microRNA in cancers, focal adhesion, and apopotosis (Figure 5E). In the above “virtual studies” results, as VEGFA-VEGFR2 signaling pathways has been reported to participation in antiangiogenic effects of TBMS1 7, we focus on “locomotion” associated with tumor microvessels, such as tumor cell adhesion and trans-endothelial migration which were reported to be regulated by the hub genes AKT1, MAPK3 and STAT3 17-19.

### TBSM1 suppresses multiple signaling pathways associated with trans-endothelial metastasis

**Figure 6:**
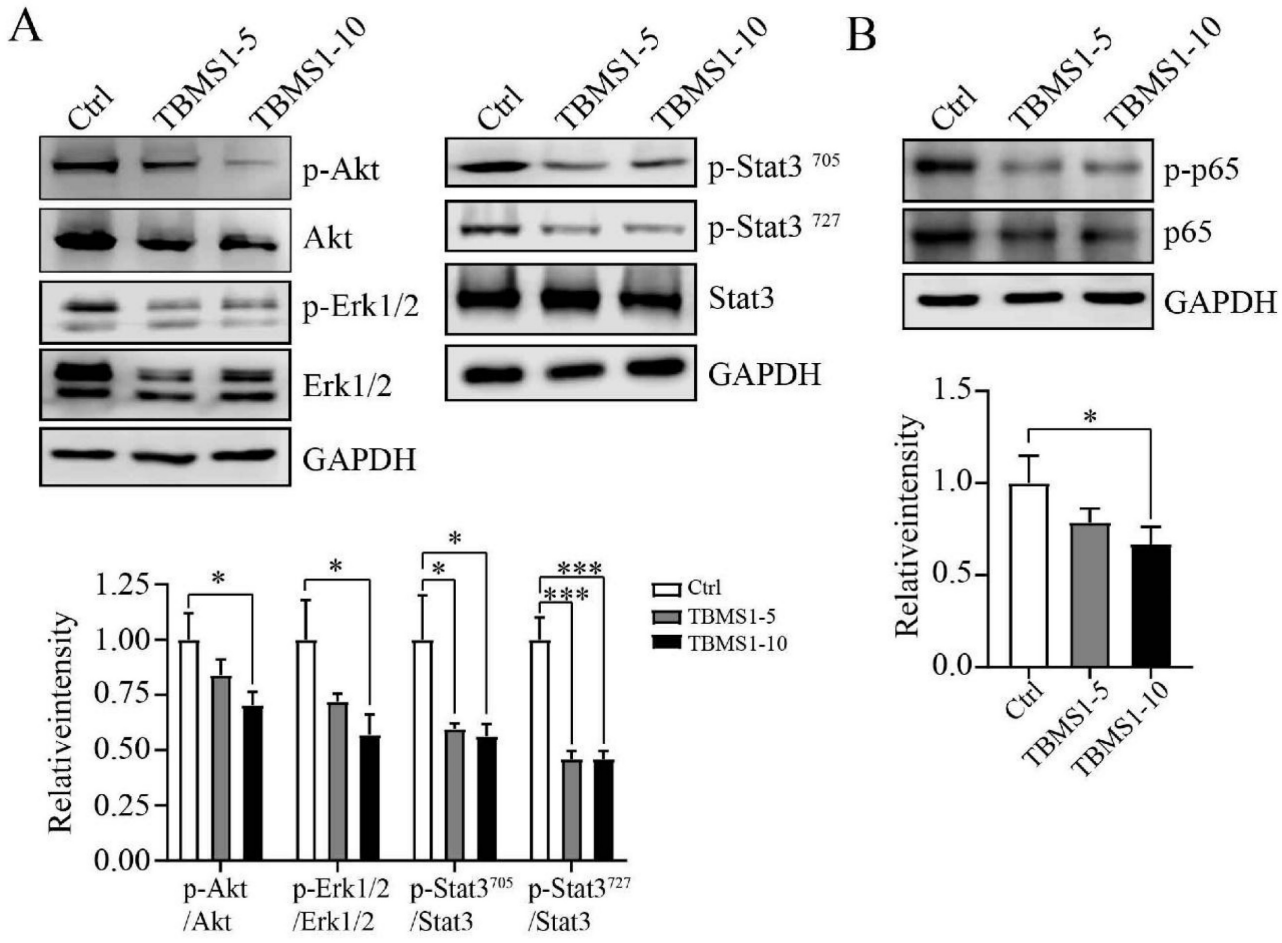
** The action of TBMS1 on signaling pathways.** (A-B) Western blot analysis of p-Akt/2/Akt, p-Erk1/2/Erk1/2 amd p-Stat3/Stat3 (A), and p-NFκB/NFκB (B) expressions were analyzed in HUVEC cells after exposure to TBSM1 (5, 10 μg/mL) for 48 h. Representative images and the summary data are shown. Data are presented as mean ± SEM of at least three independent experiments. *p < 0.05, **p < 0.01, ***p < 0.001.

Based on network pharmacological data, the protein phosphorylation of Akt, Erk1 and Stat3 coded by the interacting hub genes AKT1, MAPK3 and STAT3 were detected by western blotting. In our research, TBMS1 treatment significantly reduced the activation of Akt and Stat3 both at tyrosine 705 and serine 727, and reduced activation of Eek1/2 in the MAPK pathway (Figure 6A). Nuclear factor κB (NFκB) signaling activation in VEC was related to cancer cell adhesion 13, 14, and was found to be reduced by TBMS1 treatment (Figure 6B). These results suggest that TBMS1 inhibited abnormal activity and function of tumor endothelial cell by interfering in the activation of multiple signaling pathways that are critical for angiogenesis. The phosphorylation reduction of Akt and Erk is dose-dependent while Stat3 and p65 are not, perhaps suggest that Akt and Erk singling pathway were more sensitive with TBMS1 treatment than Stat3 and p65 singling proteins.

## Discussion

Tumor vessels are fundamental for tumor progression and metastatic dissemination 20. Inhibiting angiogenesis is a therapeutic strategy for solid tumors, and strategies including monoclonal antibodies targeting VEGF or VEGFR and small-molecule tyrosine kinase inhibitors that inhibit multiple angiogenic and proliferative pathways are approved for clinical use in a variety of cancer contexts 21, 22. Components of traditional Chinese medicine (TCM), have been shown to have strong anti-angiogenic activity 5, 23, 24. TBMS1 is an active compound form of the Chinese medicinal herb *Bolbostemma paniculatum*, and has been shown to have anti-cancer activity including ovarian cancer 25. Although it's reported that TBMS1 inhibited tumor angiogenesis by regulating of angiogenesis-related growth factors and their receptors 7, the anti-tumor vessels effectiveness of TBMS1 has not been thoroughly investigated. In the present study, we used network pharmacology and experimental validation to reveal that TBMS1 demonstrated novel anti-tumor microvessels potential by inhibiting tumor adhesion and vascular permeability.

The formation of tumor microvessels is a complex process and depends on endothelial cell migration, proliferation, and capillary tube formation 26. We firstly explored the effects and possible mechanisms of TBMS1 on vascular endothelial cells (VECs), using *in vivo* and *in vitro* models. In a SKOV3 ovarian cancer model, tumor angiogenesis suppression was more significantly than growth retardation after systemic treatment of TBMS1 (Figure 1). Meanwhile, we cultured VECs with TBMS1, and found that TBMS1 exhibited anti-angiogenic activity by inhibiting the pro-angiogenic properties of VECs, including blockade of hyper-proliferation via cell cycle arrest and increased apoptosis, decreased migration, and reduced formation of capillary structures (Figure 2-3).

Next, the PPI network analysis showed that the hub targets of TBMS1 were mainly protein kinase family members (AKT, VEGFA, EGFR, JUN, MAPK3 and STAT3). The enrichment analyses for intersection targets showed several essential biological processes and signaling pathways in tumor microvessels, including VEGFA-VEGFR2 signaling pathways, regulation of locomotion and phosphorylation, and focal adhesion, underscoring typical “multi-ingredient, multi-target, and multi-function” pharmacological characteristics of TBMS1. Based on the integrative analysis of hub targets and GO-BP/KEGG analysis, we focused on the process of adhesion and the subsequent trans-endothelial migration (Figure 4).

The surface of endothelial are covered with adhesion molecules, including ICAM-1 and VCAM-1, which mediate the adhesion and extravasation of cancer cells 11. In this study, we found that TBMS1 treatment lowered VEC expression of cancer adhesion molecules (ICAM-1 and VCAM-1), and reduced cancer cells adhesion on VEC monolayers (Figure 5). Additionally, given the transcription of these adhesion molecules was driven predominantly by the proinflammatory transcription factor NF-κB 14, phosphorylated p65 was inhibited by TBMS1 treatment (Figure 6). Additionally, VECs were strongly connected through adherent junctions, where VE-cadherin was of vital importance for the maintenance and control of endothelial cell contacts 27, and the initial assessments of TJ proteins, such as ZO-1 and claudin-5 suggested a tumor-suppressive role, with loss/reduction resulting in increased metastasis 12, 16. Here, we found that TBMS1 treatment enhanced the expression of these adherent junction proteins to reduce the vascular permeability of endothelial monolayers, as determined by evaluating the passage of dextran (Figure 5). Finally, the hub proteins phosphorylation of Akt, Erk1/2 and STAT3, which were reported to regulate adhesion and the subsequent trans-endothelial migration process, were found to be decreased after TBMS1 treatment (Figure 5). These results suggest that TBMS1 reduces tumor microvessels by interfering in the activation of multiple signaling pathways that are critical for angiogenesis.

Although multiple molecular and a variety of pathways have been recognized as possible targets of TBSM1^6^, the precise binding targets of TBSM1 using proteome microarray, co-immunoprecipitation, and other assays has not been reported and would be clarified by our further studies.

Finally, a study was shown that TBMS I promoted angiogenesis via activation of eNOS-VEGF signaling pathway and acted as a novel agent for therapeutic angiogenesis in ischemic diseases 28, which is contradictory with our research. However, this apparent paradox is neither unique to TBMS1, nor unexplained. The dual activity is frequently seen with natural drugs 29. Therefore, it is not surprising to observe that TBMS1 was found to promote angiogenesis at low concentration (0.5-2 μM) in normal tissues, but to trigger anti-angiogenesis at high concentration (5 and 10 μg/mL, equal to 3.79 and 7.58 μM) in tumor tissue. Together, TBMS1 can been acted as well-modulators of angiogenesis homeostasis.

## Conclusion

Our study proposes that TBMS1 suppresses endothelial cells' abnormal activity and function involved in cancer cell adhesion and tumor vascular permeability. These findings suggest that TBMS1 might be a powerful inhibitor of tumor microvessels by suppressing tumor adhesion and vascular permeability, ultimately reducing tumor growth and metastasis.

## Supplementary Material

Supplementary tables.
