# Supplementary material for: Unraveling the Action Mechanism of Tubeimoside-1 against Tumor Microvessels via Network Pharmacology and Experimental Validation
Source: J Cancer. 2024 Jan 1;15(4):955–65. doi: 10.7150/jca.90391 (PMC10788730; doi:10.7150/jca.90391)
Supplement: Supplementary file 1 — Supplementary tables. [file jcav15p0955s1.pdf]

Supplementary Data

Unraveling the action mechanism of Tubeimoside-1 against tumor microvessels via network pharmacology and experimental validation

Supplementary Table

Table S1. Primer sequences used in the real-time PCR assays.

Target	sequence (5'-3')	Size (bp)
CDH5	GTTCACGCATCGGTTGTTCAA	
	CGCTTCCACCACGATCTCATA	238
TJP1	ATGGAGGAAACAGCTATATGGGA	
	CCAAATCCAAATCCAGGAGCC	68
CLDN5	CTCTGCTGGTTCGCCAACAT	
	CAGCTCGTACTTCTGCGACA	75
VCAM-1	TTTGACAGGCTGGAGATAGACT	
	TCAATGTGTAATTTAGCTCGGCA	173
ICAM-1	ATGCCCAGACATCTGTGTCC	
	GGGGTCTCTATGCCCAACAA	112
	GAAGGTGAAGGTCGGAGT	
GAPDH	CATGGGTGGAATCATATTGGAA	155

Table S2. Common target genes of TBMS1and tumor microvessel.

NO.	Uniport ID	Gene	Description
1	O95342	ABCB11	Bile salt export pump
2	P33527	ABCC1	Multidrug resistance-associated protein 1
3	P12821	ACE	Angiotensin-converting enzyme
4	P22303	ACHE	Acetylcholinesterase
5	O14672	ADAM10	Disintegrin and metalloproteinase domain-containing protein 10
6	P78536	ADAM17	Disintegrin and metalloproteinase domain-containing protein 17
7	P29274	ADORA2A	Adenosine receptor A2a
8	P29275	ADORA2B	Adenosine receptor A2b
9	P07550	ADRB2	Beta-2 adrenergic receptor
10	P15121	AKR1B1	Aldo-keto reductase family 1 member B1
11	P31749	AKT1	RAC-alpha serine/threonine-protein kinase
12	Q9UM73	ALK	ALK tyrosine kinase receptor
13	P05186	ALPL	Alkaline phosphatase, tissue-nonspecific isozyme
14	Q16853	AOC3	Membrane primary amine oxidase
15	P05067	APP	Amyloid-beta precursor protein
16	P10275	AR	Androgen receptor
17	P56817	BACE1	Beta-secretase 1
18	Q07812	BAX	Apoptosis regulator BAX

19	P06276	BCHE	Cholinesterase
20	P10415	BCL2	Apoptosis regulator Bcl-2
21	Q07817	BCL2L1	Bcl-2-like protein 1
22	P15056	BRAF	Serine/threonine-protein kinase B-raf
23	O60885	BRD4	Bromodomain-containing protein 4
24	P00918	CA2	Carbonic anhydrase 2
25	P22748	CA4	Carbonic anhydrase 4
26	Q16790	CA9	Carbonic anhydrase 9
27	Q16602	CALCRL	Calcitonin gene-related peptide type 1 receptor
28	P07384	CAPN1	Calpain-1 catalytic subunit
29	P42574	CASP3	Caspase-3
30	Q14790	CASP8	Caspase-8
31	P32239	CCKBR	Gastrin/cholecystokinin type B receptor
32	P24385	CCND1	G1/S-specific cyclin-D1
33	P24864	CCNE1	G1/S-specific cyclin-E1
34	P41597	CCR2	C-C chemokine receptor type 2
35	P51681	CCR5	C-C chemokine receptor type 5
36	P24941	CDK2	Cyclin-dependent kinase 2
37	P11802	CDK4	Cyclin-dependent kinase 4
38	P21554	CNR1	Cannabinoid receptor 1
39	Q92793	CREBBP	CREB-binding protein
40	P07858	CTSB	Cathepsin B
41	P07339	CTSD	Cathepsin D
42	P14091	CTSE	Cathepsin E
43	P25024	CXCR1	C-X-C chemokine receptor type 1
44	P25025	CXCR2	C-X-C chemokine receptor type 2
45	Q9NS75	CYSLTR2	Cysteinyl leukotriene receptor 2
46	P27487	DPP4	Dipeptidyl peptidase 4
47	P25101	EDNRA	Endothelin-1 receptor
48	P24530	EDNRB	Endothelin receptor type B
49	P00533	EGFR	Epidermal growth factor receptor
50	P08246	ELANE	Neutrophil elastase
51	P04626	ERBB2	Receptor tyrosine-protein kinase erbB-2
52	P03372	ESR1	Estrogen receptor
53	Q92731	ESR2	Estrogen receptor beta
54	P00742	F10	Coagulation factor X
55	P03951	F11	Coagulation factor XI
56	P00734	F2	Prothrombin
57	P55085	F2RL1	Proteinase-activated receptor 2
58	P13726	F3	Tissue factor
59	P00740	F9	Coagulation factor IX
60	O14842	FFAR1	Free fatty acid receptor 1
61	P05230	FGF1	Fibroblast growth factor 1
62	P09038	FGF2	Fibroblast growth factor 2

63	P11362	FGFR1	Fibroblast growth factor receptor 1
64	P17948	FLT1	Vascular endothelial growth factor receptor 1
65	P08151	GLI1	Zinc finger protein GLI1
66	Q05586	GRIN1	Glutamate receptor ionotropic, NMDA 1
67	Q12879	GRIN2A	Glutamate receptor ionotropic, NMDA 2A
68	Q13224	GRIN2B	Glutamate receptor ionotropic, NMDA 2B
69	P49841	GSK3B	Glycogen synthase kinase-3 beta
70	Q13547	HDAC1	Histone deacetylase 1
71	Q9Y251	HPSE	Heparanase
72	P25021	HRH2	Histamine H2 receptor
73	P07900	HSP90AA1	Heat shock protein HSP 90-alpha
74	P28222	HTR1B	5-hydroxytryptamine receptor 1B
75	P28221	HTR1D	5-hydroxytryptamine receptor 1D
76	P28223	HTR2A	5-hydroxytryptamine receptor 2A
77	P41595	HTR2B	5-hydroxytryptamine receptor 2B
78	P46098	HTR3A	5-hydroxytryptamine receptor 3A
79	O75874	IDH1	Isocitrate dehydrogenase [NADP] cytoplasmic
80	P14902	IDO1	Indoleamine 2,3-dioxygenase 1
81	O14920	IKBKB	Inhibitor of nuclear factor kappa-B kinase subunit beta
82	P60568	IL2	Interleukin-2
83	P13612	ITGA4	Integrin alpha-4
84	P06756	ITGAV	Integrin alpha-V
85	P18084	ITGB5	Integrin beta-5
86	P05412	JUN	Transcription factor Jun
87	Q12809	KCNH2	Potassium voltage-gated channel subfamily H member 2
88	P29375	KDM5A	Lysine-specific demethylase 5A
89	P21397	MAOA	Amine oxidase [flavin-containing] A
90	Q99558	MAP3K14	Mitogen-activated protein kinase kinase kinase 14
91	P28482	MAPK1	Mitogen-activated protein kinase 1
92	Q16539	MAPK14	Mitogen-activated protein kinase 14
93	P27361	MAPK3	Mitogen-activated protein kinase 3
94	P45983	MAPK8	Mitogen-activated protein kinase 8
95	P49137	MAPKAPK 2	MAP kinase-activated protein kinase 2
96	Q07820	MCL1	Induced myeloid leukemia cell differentiation protein Mcl-1
97	Q00987	MDM2	E3 ubiquitin-protein ligase Mdm2
98	P08581	MET	Hepatocyte growth factor receptor
99	P50579	METAP2	Methionine aminopeptidase 2
100	P08473	MME	Neprilysin
101	P03956	MMP1	Interstitial collagenase
102	P08253	MMP2	72 kDa type IV collagenase
103	P14780	MMP9	Matrix metalloproteinase-9
104	P43490	NAMPT	Nicotinamide phosphoribosyltransferase
105	P19838	NFKB1	Nuclear factor NF-kappa-B p105 subunit

106	P35228	NOS2	Nitric oxide synthase, inducible
107	Q9NPH5	NOX4	NADPH oxidase 4
108	P25929	NPY1R	Neuropeptide Y receptor type 1
109	P04150	NR3C1	Glucocorticoid receptor
110	P41145	OPRK1	Kappa-type opioid receptor
111	P09874	PARP1	Poly [ADP-ribose] polymerase 1
112	O00408	PDE2A	cGMP-dependent 3',5'-cyclic phosphodiesterase
113	P06401	PGR	Progesterone receptor
114	P42336	PIK3CA	Phosphatidylinositol 4,5-bisphosphate 3-kinase catalytic subunit alpha isoform
115	P27986	PIK3R1	Phosphatidylinositol 3-kinase regulatory subunit alpha
116	P11309	PIM1	Serine/threonine-protein kinase pim-1
117	P14555	PLA2G2A	Phospholipase A2, membrane associated
118	P47712	PLA2G4A	Cytosolic phospholipase A2
119	P00749	PLAU	Urokinase-type plasminogen activator
120	Q03181	PPARD	Peroxisome proliferator-activated receptor delta
121	P37231	PPARG	Peroxisome proliferator-activated receptor gamma
122	P36873	PPP1CC	Serine/threonine-protein phosphatase PP1-gamma catalytic subunit
123	P17252	PRKCA	Protein kinase C alpha type
124	P05771	PRKCB	Protein kinase C beta type
125	Q05655	PRKCD	Protein kinase C delta type
126	Q04759	PRKCQ	Protein kinase C theta type
127	P78527	PRKDC	DNA-dependent protein kinase catalytic subunit
128	P49768	PSEN1	Gamma-secretase，Presenilin-1
129	P49810	PSEN2	Gamma-secretase，Presenilin-2
130	P25105	PTAFR	Platelet-activating factor receptor
131	P35408	PTGER4	Prostaglandin E2 receptor EP4 subtype
132	O14684	PTGES	Prostaglandin E synthase
133	P35354	PTGS2	Prostaglandin G/H synthase 2
134	Q05397	PTK2	Focal adhesion kinase 1
135	Q15257	PTPA	Serine/threonine-protein phosphatase 2A activator
136	O95136	S1PR2	Sphingosine 1-phosphate receptor 2
137	Q99500	S1PR3	Sphingosine 1-phosphate receptor 3
138	Q8IXJ6	SIRT2	NAD-dependent protein deacetylase sirtuin-2
139	Q96S37	SLC22A12	Solute carrier family 22 member 12
140	P11166	SLC2A1	Solute carrier family 2, facilitated glucose transporter member 1
141	Q01959	SLC6A3	Sodium-dependent dopamine transporter
142	P30874	SSTR2	Somatostatin receptor type 2
143	P35346	SSTR5	Somatostatin receptor type 5
144	P40763	STAT3	Signal transducer and activator of transcription 3
145	P25103	TACR1	Substance-P receptor
146	O14746	TERT	Telomerase reverse transcriptase
147	P36897	TGFBR1	TGF-beta receptor type-1
148	Q9NR96	TLR9	Toll-like receptor 9

149	P11387	TOP1	DNA topoisomerase 1
150	P04637	TP53	Cellular tumor antigen p53
151	Q15661	TPSAB1	Tryptase alpha/beta-1
152	Q9HBA0	TRPV4	Transient receptor potential cation channel subfamily V member 4
153	P11473	VDR	Vitamin D3 receptor
154	P15692	VEGFA	Vascular endothelial growth factor A
155	P47989	XDH	Xanthine dehydrogenase/oxidase
